# Implications of Hemostasis Disorders in Patients with Critical Limb Ischemia—An In-Depth Comparison of Selected Factors

**DOI:** 10.3390/jcm9030659

**Published:** 2020-02-29

**Authors:** Radosław Wieczór, Arleta Kulwas, Danuta Rość

**Affiliations:** 1 Department of Pathophysiology, Faculty of Pharmacy, Nicolaus Copernicus University in Toruń, Ludwik Rydygier Collegium Medicum in Bydgoszcz, 85–094 Bydgoszcz, Poland; arletakulwas@wp.pl (A.K.); drosc@cm.umk.pl (D.R.); 2 Clinic of Vascular and Internal Medicine, Dr Jan Biziel University Hospital No. 2 in Bydgoszcz, 85-168 Bydgoszcz, Poland

**Keywords:** critical limb ischemia, hemostasis, biomarkers

## Abstract

Background: Atherosclerosis is a systemic disease. Among patients with atherosclerosis, those suffering from peripheral arterial disease (PAD) represent a group of individuals with particularly high death risk, especially during the course of critical limb ischemia (CLI). In the pathogenesis of PAD/CLI complications, blood coagulation disorders play a significant role. The study aim was to examine the activation of the coagulation system depending on tissue factor (TF) in patients with CLI as compared with those with intermittent claudication (IC). Methods: Before initiating proper treatment (invasive or maintenance), blood samples were collected from 65 patients with CLI and 15 with IC to measure the following selected hemostasis parameters: concentrations and activation of tissue factor (TF Ag and TF Act) and tissue factor pathway inhibitor (TFPI Ag and TFPI Act), concentrations of thrombin–antithrombin complex (TAT Ag) and fibrinogen, platelet count (PLT), and concentrations of tissue-plasminogen activator (t-PA Ag), plasminogen activator inhibitor 1 (PAI-1), and D-dimer. The control group included 30 healthy volunteers (10 female/20 male). Results: The values of all analyzed parameters (except for lower TFPI Act) were significantly higher in the blood of PAD patients (with respect to PLT only in the CLI subgroup) in comparison with healthy subjects. The blood of patients with CLI as compared to the IC subgroup revealed much higher concentrations of TF Ag (*p* < 0.001), with slightly decreased TF Act, significantly lower concentrations of TFPI Ag (*p* < 0.001), slightly increased TFPI Act, and significantly higher levels of TAT Ag (*p* < 0.001), fibrinogen (*p* = 0.026), and D-dimer (*p < 0.05*). Conclusions: In patients with CLI, we can observe coagulation activation and a shifting balance toward prothrombotic processes. Furthermore, increased concentrations of D-dimer suggest a secondary activation of fibrinolysis and confirm the phenomenon as a prothrombotic condition with heightened fibrinolysis.

## 1. Introduction

Cardio-vascular diseases (CVD) are a major cause of death in many countries including Poland. Hemostasis disorders are significant in the pathogenesis of arteriosclerosis, including in arteries of the lower limbs—i.e., in peripheral arterial disease (PAD)—with an adverse impact on prognosis which affects an increasing percentage of patients [1,2]. Also, research to date suggests that PAD might affect a greater proportion of women than men in low-income and middle-income countries [3]. The activation of the coagulation system on fibrous plaques plays a major role in the different stages of atherosclerotic development. Patients with PAD have a heightened risk for cardio-vascular events because of the systemic nature of atherosclerosis [4]. Potentially dangerous complications of advanced atherosclerotic plaques may not only mean such clinical conditions as unstable angina, myocardial infarction, sudden cardiac death, ischemic stroke, and chronic or acute peripheral arterial disease, but in particular critical limb ischemia (CLI). PAD symptoms are triggered by stenosis precipitated by an atherosclerotic plaque or arterial occlusion caused by thrombus formation on a ruptured atherosclerotic plaque. Intermittent claudication (IC) is the most typical symptom; however, IC is estimated to affect about one-third of all patients with PAD [5]. The adaptation of chronically undersupplied tissue manifests in the formation of a collateral network [6]. Limb ischemia is manifested by walking disorders, i.e., pain, fatigue, or muscle contraction, occurring after a certain distance in the buttocks, thighs, calves, or feet, with relief during rest [7]. Angiogenesis, the growth of small vessels from pre-existing vessels, and arteriogenesis, the dilation of the lumen of the pre-existing ones, may develop a collateral network [8]. Rest pain in distal areas like limbs and feet and poorly healing leg wounds (with proven ischemic etiology) are among the most severe forms of PAD, i.e., CLI. Rest pain usually lasts more than two weeks, and is associated with ischemia and common cutaneous lesions and necrosis [5]. In CLI the biological adaptations become overburdened and collateral supply is no longer sufficient to compensate for tissue hypoperfusion [6,9]. Patients with PAD and CLI have some of the highest death risk indicators [3,4,10,11]. PAD complications may also include acute limb ischemia (ALI), posing a direct risk of limb loss or even to life [12].

Clot formation on a ruptured atherosclerotic plaque in the blood vessel depends on tissue factor (TF). A rupture or ulcer (erosion) of an unstable plaque (rich in lipids, with a thin collagen layer) is the place of thrombus formation, which may lead to a partial or complete vessel occlusion [13]. Released from a damaged vessel, TF gains access to proconvertin (factor VII in plasma), with which it forms a the TF-VII complex. Factor VII, on the other hand, becomes active (VIIa) thus activating the subsequent plasma factors (VIII, IX, X) and platelets (PLTs) in the tenase complex. In the prothrombinase complex, prothrombin is converted into thrombin, which then transforms fibrin from fibrinogen [14].

The strongest natural inhibitor of the extrinsic coagulation system is tissue factor pathway inhibitor (TFPI). Anticoagulant activity is also exerted by antithrombin (AT)—a glycoprotein—produced by endothelial cells which is considered to play a key role among endothelial coagulation inhibitors. Antithrombin inactivates thrombin, binding it to a stable inactive thrombin–antithrombin complex (TAT). Increased levels of thrombin–antithrombin complex show that the coagulation system is activated in cardio-vascular diseases. TAT complexes are some of the most sensitive and specific biomarkers for thrombin formation. An increase in their concentrations reflects thrombinogenesis intensity in vivo [15,16]. The role of TF and TFPI in angiogenic processes is not fully understood, and AT is considered to be a strong inhibitor of angiogenesis [17].

The study aim was to compare selected hemostasis parameters in PAD patients in IC and CLI groups.

## 2. Material and Methods

### 2.1. Research Subjects

Within the study group of 80 patients with diagnosed PAD (27 females/53 males, average age 63.5 ± 9 years), the IC (*n* = 65) and CLI (*n* = 15) subgroups were identified. The material used in the research—venous blood—was sampled in the morning before the administration of planned treatment. Then, 45 patients were qualified for invasive treatment (percutaneous transluminal angioplasty (PTA) procedure) and 35 patients for maintenance (non-invasive) treatment (pharmacotherapy, walking training, and further observation). The control group comprised 30 healthy non-smoking subjects (10 female/20 male) with an average age of 56 ± 6 years.

Exclusion criteria, in addition to the lack of consent, cancer, and pregnancy, were as follows: acute cardio-vascular incidents during the last six months in the form of myocardial infarction/unstable angina and stroke/transient ischemic attacks, as well as intake of drugs (apart from acetylsalicylic acid and statin) affecting the hemostasis system (anticoagulants), decompensated diabetes with retinopathy, severe renal failure, chronic obstructive pulmonary diseases, and systemic diseases. 

In both groups, after giving informed consent to participate in the research, venous blood samples were collected to determine: TF Ag concentrations using the ELISA method (Imubind Tissue Factor, Sekisui®, Burlington, Massachusetts, USA), TF activity (Actichrome TF, American Diagnostica®, Stamford, Connecticut, USA), concentrations of TFPI Ag (Imubind TFPI ELISA kit, Sekisui®, Burlington, Massachusetts, USA), TFPI activity (Actichrome TFPI, American Diagnostica®, Stamford, Connecticut, USA), concentrations of TAT complex (Enzygnost TAT, Siemens®, Malvern, Pennsylvania, USA), fibrinogen concentrations using CC-3003 apparatus (Bio-ksel®, Grudziądz, Poland), PLT count with XT-4000i apparatus (Sysmex®, Kobe, Japan), and levels of tissue-plasminogen activator (t-PA Ag) by means of the ELISA method (Diagnostica Stago®, Parsippany, New Jersey, USA). Plasminogen activator inhibitor 1 (PAI-1 Ag, Sekisui Diagnostics®, Burlington, Massachusetts, USA), and D-dimer concentrations were measured using CC-3003 apparatus (Bio-ksel®, Grudziądz, Poland).

### 2.2. Statistical Analysis

Statistica 12.0 (StatSoft^®^, Cracow, Poland) was used for the statistical analysis and the significance level was set at *p* < 0.05. The compatibility of examined parameters distribution with the standard normal distribution was assessed by the Shapiro–Wilk test. For variables with abnormal distribution, non-parametric tests were applied (Mann–Whitney U test, Kruskal–Wallis test), and parametric tests were used for normal distribution (*t*–Student test, RIR Tukey’s test). The local Bioethics Commission gave consent (KB 509/2011, 2011 11 Oct) to conduct the research, and the study was carried out in accordance with the Declaration of Helsinki.

## 3. Results

The characteristics of the study group is displayed in Table 1. Most of the subjects included male subjects (two-thirds of individuals) and IC patients (*n* = 65). All patients were administered statin and acetylsalicylic acid (75 mg/day)—medication with a proven influence on the reduction of TF concentrations [18,19].

Table 2 compares the values of parameters examined in patients with IC and CLI against those of the control group.

We found that TF Ag concentrations were several times higher in both subgroups than in the control group. These levels were six times higher in patients with IC and seven times higher in the CLI subgroup. CLI patients had significantly elevated TF Ag concentrations as compared to the IC subgroup. The comparison of TF Act levels in both subgroups with healthy subjects suggested that TF Act levels were nearly six times higher in the IC group and four times higher in the CLI group when compared to the control group. However, no difference regarding TF Act was observed between these subgroups. TFPI Ag levels were significantly elevated in the IC group as compared to the CLI group and higher than in the control group (in both cases *p* < 0.001). TFPI Act were significantly lower in both subgroups of patients than in the control group (respectively *p* < 0.001 and p < 0.001). Concentrations of TAT complex were 24 times higher in the IC group and 30 times in the CLI group than in the control group. A comparison of TAT complex concentrations in both groups demonstrates significantly higher concentrations of TAT in CLI patients than in IC subjects. The analysis of fibrinogen concentration shows it was the highest in the CLI group, significantly higher than in the IC group (*p* = 0.026), and higher than in the control group *(p* < 0.001). A significantly elevated PLT count was observed in the CLI group as compared to the control group (*p* = 0.025).

We also measured key fibrinolysis parameters, that is, the concentrations of t-PA Ag, PAI-1 Ag, and D-dimer. In both groups of patients, concentrations of t-PA Ag, PAI-1 Ag, and D-dimer were significantly higher than in the control group (*p* < 0.001 in all cases). D-dimer levels were significantly higher in the CLI group than in the IC group (*p* < 0.05).

## 4. Discussion

The study revealed that the concentrations of analyzed parameters were higher in both groups of patients with PAD (IC and CLI) than in the control group (except for TFPI Act). The comparison of patients with IC and CLI shows significantly heightened levels of TF Ag, TAT complexes, fibrinogen, and D-dimer, and lower concentrations of TFPI Ag in CLI patients when compared to healthy subjects. 

Elevated TF levels in PAD cases were observed by Blann et al. in 42 patients [20] and Makin et al. [21]. Kotschy et al., in turn, emphasized the role of high concentrations of TF and fibrinogen in restenosis following revascularization procedures [22]. Endothelial cells activated in the course of the disease have increased secretion of pro-inflammatory cytokines and production of adhesion molecules. This, in turn, triggers the growth of immune cells in atherosclerotic regions [23]. Penetration of macrophages and lymphocytes into damaged vessels increases and destabilizes the atherosclerotic plaque [24,25]. The continued decrease in the flow characterizes IC and destabilized plaque results in its rupture and thrombus formation in the place of plaque, damage which is the main cause of CLI, with collateral circulation poorly developed in angio- and arteriogenesis. The consequences include vessel occlusion and hypoxia of affected regions.

In the pathogenesis of atherosclerosis and its complications, platelets (PLTs) play a special role mainly due to their prothrombic potential influenced by the presence of numerous plasma hemostasis factors in cytoplasmic granules. In atherosclerosis, including PAD, a significant change can be observed in their morphology, function and activation degree which results not only in progression of the disease, but first of all in life-threatening complications including CLI [26]. Although no significant differences in PLT count were determined between patients with IC and CLI, PLT count was significantly increased in patients with CLI as compared to healthy subjects. Recently, with regard to thrombotic complications in patients with PAD, the so-called platelet microparticles (PMPs) have been considered to be of importance. High levels of PMPs were reported by Tan et al. [27]. These authors showed that these microparticles contain significant amounts of TF, which in turn is considered to be the key factor activating the coagulation system. Damaged endothelium provides an extra source of TF which in consequence activates monocytes/macrophages, leucocytes, and platelets.

The research constraints included the analysis of only one platelet parameter, namely, the assessment of the absolute count. The analysis did not cover activation, aggregation (also mean platelet volume, MPV), or other significant platelet biomarkers released from granulation. Increased platelet activation has been already reported in many studies on patients with PAD [28,29,30,31,32,33,34]. Considering the fact that each platelet engaged in coagulation improves the prothrombic potential in the vessel lumen, an increased platelet count in patients with atherosclerosis comprises a significant element of thrombotic risk in these patients, and this relatively uncomplicated and common test can prove to be extremely valuable in the diagnostics of PAD patients.

TF-dependent coagulation activation remains under the control of TFPI. In the present study, the concentrations of TFPI Ag were significantly lower in CLI patients than in the IC subgroup. Lower TFPI Ag levels in patients with PAD were reported by Blann et al. [20]. As in this study, Radziwon et al. recorded elevated concentrations of TFPI in patients with IC [35]. It is believed that 70–80% TFPI remains bound to endothelium and only 20–30% circulates unbound [36]. In our patients the TFPI Act levels were decreased in both groups of patients with PAD, i.e., IC and CLI. Therefore, reduced TFPI Act was determined in patients with PAD and CLI, with lower concentrations than in IC patients and lower activation than in healthy subjects. 

Increased concentrations of TAT complex demonstrate intensified generation of thrombin in cardiovascular diseases, and thus it is one of the most sensitive thrombosis biomarkers, but is not very specific. In the present study, elevated concentrations of TAT significantly differentiated patients with CLI as compared to IC. Increased concentrations of TAT were reported by Strano et al., Hering et al., and Cassar et al. [37,38,39]. As illustrated in the group with CLI, thrombinogenesis is stronger than in IC patients, corresponding with increased formation of TF in this group in the course of activation of the so-called extrinsic coagulation pathway.

A compensatory mechanism in relation to hypercoagulability is the fibrinolysis system which, for long periods of time, can protect the body against clinically evident thrombosis. Endothelial t-PA present in circulating blood leads to the formation of plasmin from plasminogen. This strong proteolytic enzyme disintegrates the fibrin which is formed during coagulation, and is a component of micro clots that appear on the damaged endothelial layer. The activation of fibrinolysis induced by plasmin is still controlled by PAI-1 released from endothelium and platelets. For a better understanding of homeostasis changes in patients with PAD, primary fibrinolysis parameters were tested, i.e., t-PA Ag, PAI-1 Ag, and D-dimer. In the present study, we observed increased concentrations of t-PA Ag and PAI-1 Ag with a simultaneous increase in D-dimer concentration. In this research no significant differences regarding these parameters were found between subgroups of patients with IC and CLI, but significantly higher mean concentrations of D-dimer were noted. In the blood of 40 patients with critical limb ischemia (CLI), Pärssons et al. determined significantly higher concentrations of t-PA and D-dimer when compared to healthy subjects [40]. Yet, higher values of t-PA and PAI-1 were observed in patients with CLI by Chudý et al. [41]. Also, Park et al. observed that blood levels of t-PA tended to increase with age in 103 patients with CLI [42].

The sources quoted above indicated a certain process occurring in patients with atherosclerosis, namely, the coexistence of intensified fibrinolysis (as demonstrated by high values of D-dimer) and fibrinolysis inhibition (due to high concentrations of PAI-1), and simultaneous elevated t-PA Ag levels. Increased concentrations of t-PA Ag are the result of intensified release from damaged endothelium as a response to a prothrombotic condition in blood occurring in atherosclerosis. High concentrations of PAI-1 Ag in atherosclerosis are a compensatory mechanism in relations to excessive release of t-PA. Increased D-dimer concentrations suggest secondary activation of fibrinolysis. This phenomenon is often referred to as “the prothrombotic condition with increased fibrinolysis” [40].

In conclusion, it should be highlighted that elevated concentrations of TF and fibrinogen observed in the blood of patients with CLI suggest prothrombic intensification. In patients with PAD and CLI, the coagulation system is activated, which manifests with higher concentrations of TAT complex when compared to subjects with IC. Higher concentrations of TF and simultaneous inhibition of TFPI activity may be decisive in the development of thrombotic complications in the course of CLI. Elevated concentrations of D-dimer indicate a secondary activation of fibrinolysis.

## 5. Limitations of the Study

Numerous factors are involved in the hemostatic process. The examination of the other factors, e.g., platelets coagulation (e.g., Annexin V) or inflammation factors (like Interleukin 6) may provide an interesting complement to our research. In the present study we chose the most significant factors for CLI and those important in clinical practice (e.g., t-PA, TF Ag, D-dimer, and fibrinogen). One of the limitations of the study is the low number of subjects studied. However, as we have demonstrated in the discussion section, a fairly large number of studies dealing with CLI involve numbers which are similar to our study and sometimes even smaller. Considering the complex nature of hemostasis disorders in PAD, further research is still required.

## 6. Conclusions

The blood of patients with CLI provides hemostatic conditions which cause a predisposition towards coagulation in the form of high concentrations of TF and fibrinogen, with a lower inhibition response of TFPI than in IC patients. Intensive thrombinogenesis in patients with CLI provides indirect evidence of hypercoagulation. Increased concentrations of D-dimer in CLI patients suggest activation of fibrinolysis secondary to excessive blood coagulation.

## Figures and Tables

**Table 1:** General characteristics of the study group (PAD, *n = 80*). PAD: peripheral arterial disease.

Parameter	Unit	Value
Sex (females/males)	*n* (%)/*n* (%)	27 (34%)/53 (66%)
Mean age ± SD	years	63.5 ± 9
Patients with intermittent claudication (IC)	*n* (%)	65 (81%)
Patients with CLI	*n* (%)	15 (19%)
Average distance IC ± SD	meters	100 ± 87
Average value ABI ± SD	( )	0.5 ± 0.25
Patients with ASA intake	*n* (%)	80 (100%)
Patients with statin intake	*n* (%)	80 (100%)
Mean concentration of LDL ± SD	mg/dL	119.7 ± 39.3
Patients with type 2 diabetes	*n* (%)	28 (35%)
Smokers (all, past)	*n* (%)	74 (92.5%)
Current smokers	*n* (%)	27 (34%)
Patients eligible for endovascular therapy (PTA)	*n* (%)	45 (56%)
Patients eligible for non-invasive (NI) treatment	*n* (%)	35 (44%)

SD: standard deviation; IC: intermittent claudication; CLI: critical limb ischemia; ABI: ankle-brachial index; ASA: acetylsalicylic acid; LDL: low-density lipoprotein; PTA: percutaneous transluminal angioplasty.

**Table 2:** Values of parameters analyzed in the study group within the subgroups of patients with intermittent claudication (IC) and critical limb ischemia (CLI) against the control group.

Parameter and Unit	Value	Study Group (PAD, *n = 80*)		Control Group (C, *n* = 30) *c*	*p*
		IC (*n = 65*) *a*	CLI (*n = 15*) *b*		
**TF Ag** (pg/mL)	X (± SD)	764.1 (± 414.4)	**877.9** (± 533.2)	133.2 (± 62.5)	*a vs b < 0.001* *a vs c < 0.001* *b vs c < 0.001*
**TF Act** (pM)	X (± SD)	24.4 (± 18.9)	15.8 (± 16.8)	4.2 (± 3.96)	*a vs b NS* *a vs c < 0.001* *b vs c < 0.001*
**TFPI Ag** (ng/mL)	X (± SD)	**102.3** (± 60.9)	83.7 (± 59.6)	71.3 (± 33.18)	*a vs b < 0.001* *a vs c < 0.001* *b vs c < NS*
**TFPI Act** (unit/mL)	X (± SD)	0.76 (± 0.53)	1.0 (± 0.5)	1.98 (± 1.01)	*a vs b NS* *a vs c < 0.001* *b vs c < 0.001*
**TAT** (ng/mL)	X (± SD)	57.7 (± 58.1)	**81.7** (± 52.1)	2.7 (± 0.9)	*a vs b = 0.02* *a vs c < 0.001* *b vs c < 0.001*
**fibrinogen** (g/L)	Me (Q25;Q75)	4.19 (3.5;4.99)	**5.33** (4.64;6.16)	3.36 (2.8;3.7)	*a vs b = 0.026* *a vs c < 0.001* *b vs c < 0.001*
**PLT** (G/L)	Me (Q25;Q75)	238 (204;273)	315 (202;369)	223 (182;282)	*a vs b NS* *a vs c NS* *b vs c = 0.025*
**t-PA Ag** (ng/mL)	Me (Q25;Q75)	13.06 (8.69;16.09)	11.96 (9.88;16.89)	4.79 (2.62;5.77)	*a vs b NS* *a vs c < 0.001* *b vs c < 0.001*
**PAI-1 Ag** (ng/mL)	Me (Q25;Q75)	50.75 (42.83;55.91)	52.66 (31.05;58.84)	16.81 (14.02;22.01)	*a vs b NS* *a vs c < 0.001* *b vs c < 0.001*
**D-dimer** (ng/mL)	X (± SD)	792.49 (± 281.17)	**966.5** (± 205.34)	312.58 (± 93.25)	*a vs b < 0.05* *a vs c < 0.001* *b vs c < 0.001*

Me: median; Q25: lower quartile; Q75: upper quartile; X: mean; SD: standard deviation; TF Ag: tissue factor concentration, TF Act: tissue factor activity; TFPI Ag: tissue factor pathway inhibitor concentration; TFPI Act: tissue factor pathway inhibitor activity; TAT: thrombin–antithrombin complex; PLT: platelet count; t-PA Ag: tissue-plasminogen activator concentration; PAI-1 Ag: plasminogen activator inhibitor 1 concentration; NS: statistically insignificant.
